# Exogenous Let-7a-5p Induces A549 Lung Cancer Cell Death Through BCL2L1-Mediated PI3Kγ Signaling Pathway

**DOI:** 10.3389/fonc.2019.00808

**Published:** 2019-08-23

**Authors:** Shuyin Duan, Songcheng Yu, Teng Yuan, Sanqiao Yao, Lin Zhang

**Affiliations:** ^1^Key Laboratory of Birth Regulation and Control Technology of National Health Commission of China, Shandong Maternal and Child Health Care Hospital, Jinan, China; ^2^School of Public Health, Zhengzhou University, Zhengzhou, China; ^3^College of Jitang, North China University of Science and Technology, Tangshan, China; ^4^School of Public Health, Xinxiang Medical University, Xinxiang, China; ^5^School of Public Health and Management, Weifang Medical University, Weifang, China

**Keywords:** lung cancer, macrophage, exosome, BCL2L1, let-7a-5p, autophagy

## Abstract

Elevated expression of let-7a-5p contributes to suppression of lung cancer, in which let-7a-5p, as exosome cargo, can be transported from macrophages to lung cancer cells, yet the role of let-7a-5p remains unclear. Utilizing bioinformatics methods and cellular experiments, this study was designed and conducted to identify let-7a-5p regulatory network in lung cancer. Bioinformatics analysis and Kaplan-Meier survival analysis revealed that let-7a-5p could directly target BCL2L1, and aberrant expression of let-7a-5p affects the survival of lung cancer patients, which was confirmed in A549 lung cancer cells using luciferase reporter assay. Moreover, let-7a-5p inhibited BCL2L1 expression and suppressed lung cancer cell proliferation, migration, and invasion. Functionally, overexpression of let-7a-5p promoted both autophagy and cell death in A549 lung cancer cells through PI3Kγ signaling pathway, whereas the apoptosis and pyroptosis of A549 lung cancer cells were unaffected. Furthermore, aberrant expression of BCL2L1 significantly altered the expression of lung cancer biomarkers such as MYC, EGFR, and Vimentin. To sum up, these data demonstrate that exogenous let-7a-5p induces A549 lung cancer cell death through BCL2L1-mediated PI3Kγ signaling pathway, which may be a useful target for lung cancer treatment.

## Introduction

Lung cancer is one of the most common respiratory diseases worldwide, for which there have been no effective treatment methods (1). With the estimated incidence number of 2,093,876 and the mortality number of 1,761,007 in 185 countries in 2018 (2), lung cancer has become the most life-threatening disease in both developed and developing countries.

The dominant role of autophagy in the proliferation, migration, and invasion of lung cancer cells has been well-established throughout previous studies (3–5). However, the mechanism underlying the initiation of autophagy is still unclear. In general, autophagy is a bulk degradation process in which organelles and cytoplasm are engulfed and transported to lysosomes for proteolysis, which is essential for the management of metabolic stress and the maintenance of normal metabolic activities (6). However, autophagy is also widely activated in various kinds of cancer cells, and certain studies demonstrated that autophagy also leads to cell death (7–9), but the role of autophagy in lung cancer is still in debate.

To unveil the role of autophagy in lung cancer, the current study was designed and conducted based on our previous work, in which we found that let-7a-5p along with its 4 target genes, including BCL2-like protein 1 (BCL2L1), insulin-like growth factor 1 receptor (IGF1R), mitogen-activated protein kinase 8 (MAPK8), and FAS, were putatively associated with the autophagy of lung cancer cells (10), and BCL2L1 was confirmed to be negatively correlated with the survival of lung cancer patients. Coincidentally, Li et al. indicated that synergistical application of hyperoside and let-7a-5p induces G1/S phase arrest and lead to suppression of lung cancer cell proliferation (11). A meta-analysis also suggests that downregulation of let-7 families corresponds to poor prognosis in patients with multiple cancers (12). Herein, let-7a-5p and its 4 target genes were recruited to investigate the role of autophagy in the carcinogenesis of lung cancer.

## Results

### Construction of Let-7a-5p Regulatory Network in Lung Cancer

**Figure 1:**
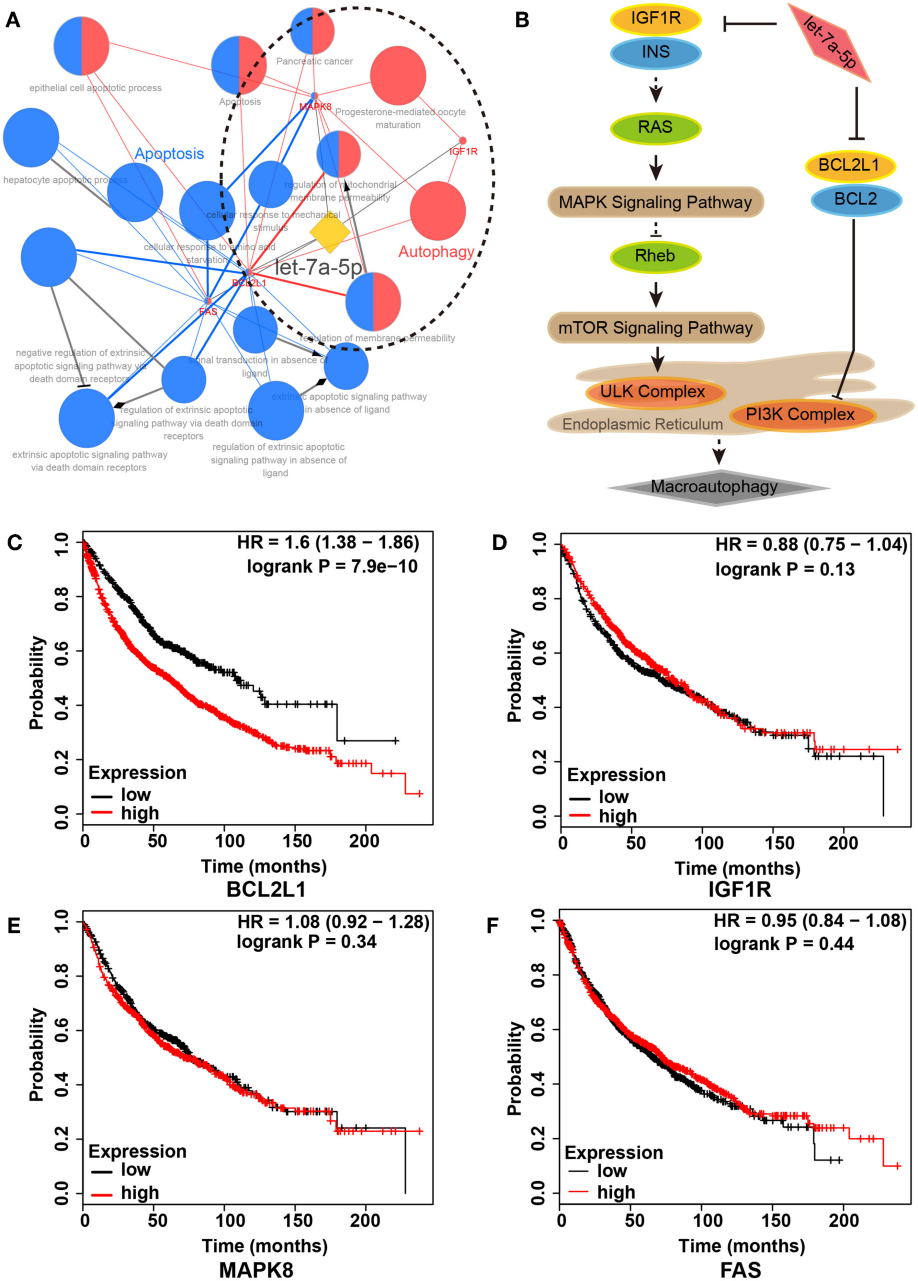
Regulatory network of let-7a-5p in lung cancer. **(A)** Functional annotation of let-7a-5p by bioinformatics analysis. Two functional groups of autophagy (Red) and apoptosis (Blue) were identified. **(B)** Schematic of autophagy regulated by let-7a-5p via targeting BCL2L1 and IGF1R in lung cancer. **(C–F)** Kaplan–Meier survival analysis in 2,437 lung cancer patients with different expressions of BCL2L1, IGF1R, MAPK8, and FAS, respectively. HR, hazard ratio.

To unveil the role of let-7a-5p in lung cancer, we conducted functional annotation for let-7a-5p using bioinformatics methods. A total of 17 GO/KEGG items were obtained, and two functional groups of autophagy and apoptosis were identified (Figures 1A,B, Table S1). Moreover, we assessed the death risk of lung cancer patients using 4 target genes related to let-7a-5p, including BCL2L1, IGF1R, MAPK8, and FAS. As shown in Figures 1C–F, high expression of BCL2L1 elevated lung cancer patients death risk [hazard ratio (HR) and 95% confidence intervals (CI) = 1.60(1.38–1.86)], but no similar correlations were found in IGF1R, MAPK8, and FAS. These data suggest that BCL2L1 is a potential target of let-7a-5p in lung cancer, and the autophagy regulated by let-7a-5p via targeting BCL2L1 is essential for the survival of lung cancer patients.

### Let-7a-5p Directly Targets BCL2L1 and Inhibits It's Expression

**Figure 2:**
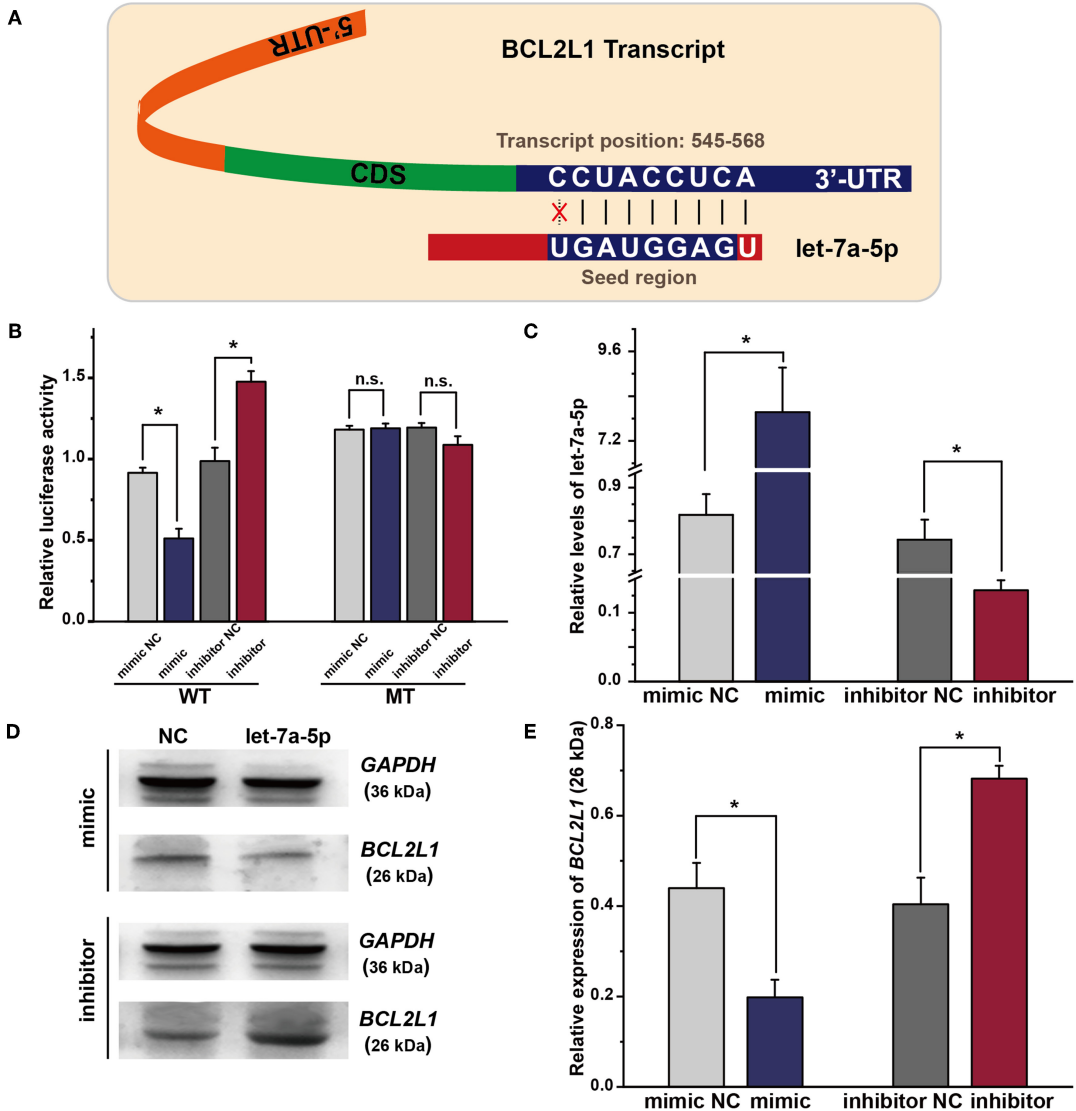
Exogenous let-7a-5p inhibits the expression of BCL2L1 in A549 lung cancer cells. **(A)** The crosstalk between let-7a-5p and BCL2L1 transcript. **(B)** The luciferase plasmid reporter assay of let-7a-5p-BCL2L1 crosstalk. The mutant (MT) and/or wild-type (WT) A549 lung cancer cells were transfected with both luciferase reporters and let-7a-5p mimics or inhibitors. **(C)** RT-qPCR assays of let-7a-5p in A549 lung cancer cells transfected with let-7a-5p mimics or inhibitors. **(D,E)** Western blot gels and protein expression analysis of BCL2L1. NC represents the negative control. ^*^P < 0.05 compared with the control group using the pooled variance *t*-test.

As predicted by bioinformatics analysis, BCL2L1 is a putative target of let-7a-5p in lung cancer. Thus, the seed region of let-7a-5p, 7 bases start from the second base at the 3′ region of let-7a-5p, was supposed to bind the 3′-UTR sequence of BCL2L1 transcript (Figure 2A). To validate the hypothesis, we adopted luciferase reporter plasmid assay by inducing mutation in the 3′-UTR region of BCL2L1, and the let-7a-5p mimics or inhibitors were co-transfected into the mutant or wild-type A549 lung cancer cells. For cells of the wild-type, the relative luciferase activity of the reporters of let-7a-5p mimics decreased comparing with the mimic control group, whereas the relative luciferase activity increased in cells transfected with let-7a-5p inhibitors. In contrast, the relative luciferase activity of the reporters of the mutant 3′-UTR region of BCL2L1 was unaffected after the cells being transfected with let-7a-5p mimics or inhibitors (Figure 2B).

To further explore the regulatory mechanism of let-7a-5p on BCL2L1, we conducted RT-qPCR and western blot to detect the expressions of let-7a-5p and BCL2L1 after the cells being transfected with let-7a-5p mimics or inhibitors, respectively. The expression of let-7a-5p increased after being transfected with let-7a-5p mimics, while it decreased after transfection of let-7a-5p inhibitors (Figure 2C). In contrast, the expression of BCL2L1 was downregulated at high level let-7a-5p, but it elevated in cells transfected with let-7a-5p inhibitors (Figures 2D,E). Consequently, our data demonstrate that let-7a-5p inhibits the expression of BCL2L1 via targeting the 3′-UTR region of BCL2L1 transcript.

### Abbarent Expression of Let-7a-5p Alters the Migration and Invasion of A549 Lung Cancer Cells

**Figure 3:**
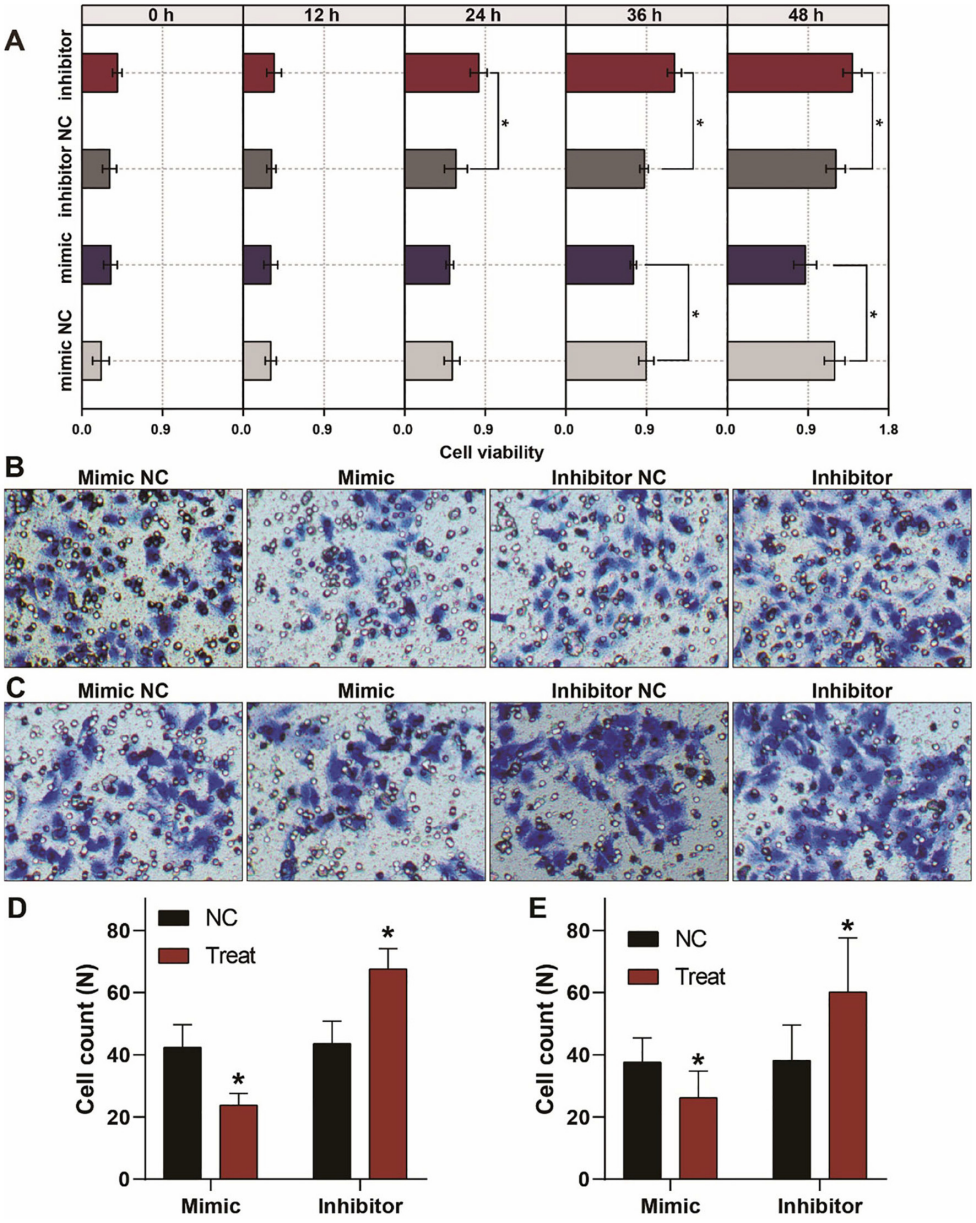
Aberrant expression of let-7a-5p alters the growth, migration, and invasion of A459 lung cancer cells. **(A)** CCK8 assays show that let-7a-5p suppresses the growth of A549 lung cancer cells. **(B,C)** Transwell assays demonstrate that let-7a-5p inhibits the invasion **(B)** and migration **(C)** of A549 lung cancer cells. **(D,E)** Quantitative analysis of **(B,C)**. NC represents the negative control. ^*^P < 0.05 compared with the control group, using the pooled variance *t*-test.

To investigate the biological behaviors of let-7a-5p on A549 lung cancer cells, we conducted CCK8 assays to evaluate the cellular viability of A549 lung cancer cells. As shown in Figure 3A, overexpression of let-7a-5p suppressed cell proliferation, while knockdown of let-7a-5p promoted cell proliferation. We then performed transwell assays to estimate the migration and invasion of A549 lung cancer cells. Knockdown of let-7a-5p significantly enhanced the migration and invasion of A549 lung cancer cells, but overexpression of let-7a-5p suppressed the cells' migration and invasion (Figures 3B–E). These data suggest that let-7a-5p can modulate the proliferation, migration, and invasion of A549 lung cancer cells, which may be crucial in the development of lung cancer.

### BCL2L1 Promotes the Proliferation, Migration, and Invasion of A549 Lung Cancer Cells

**Figure 4:**
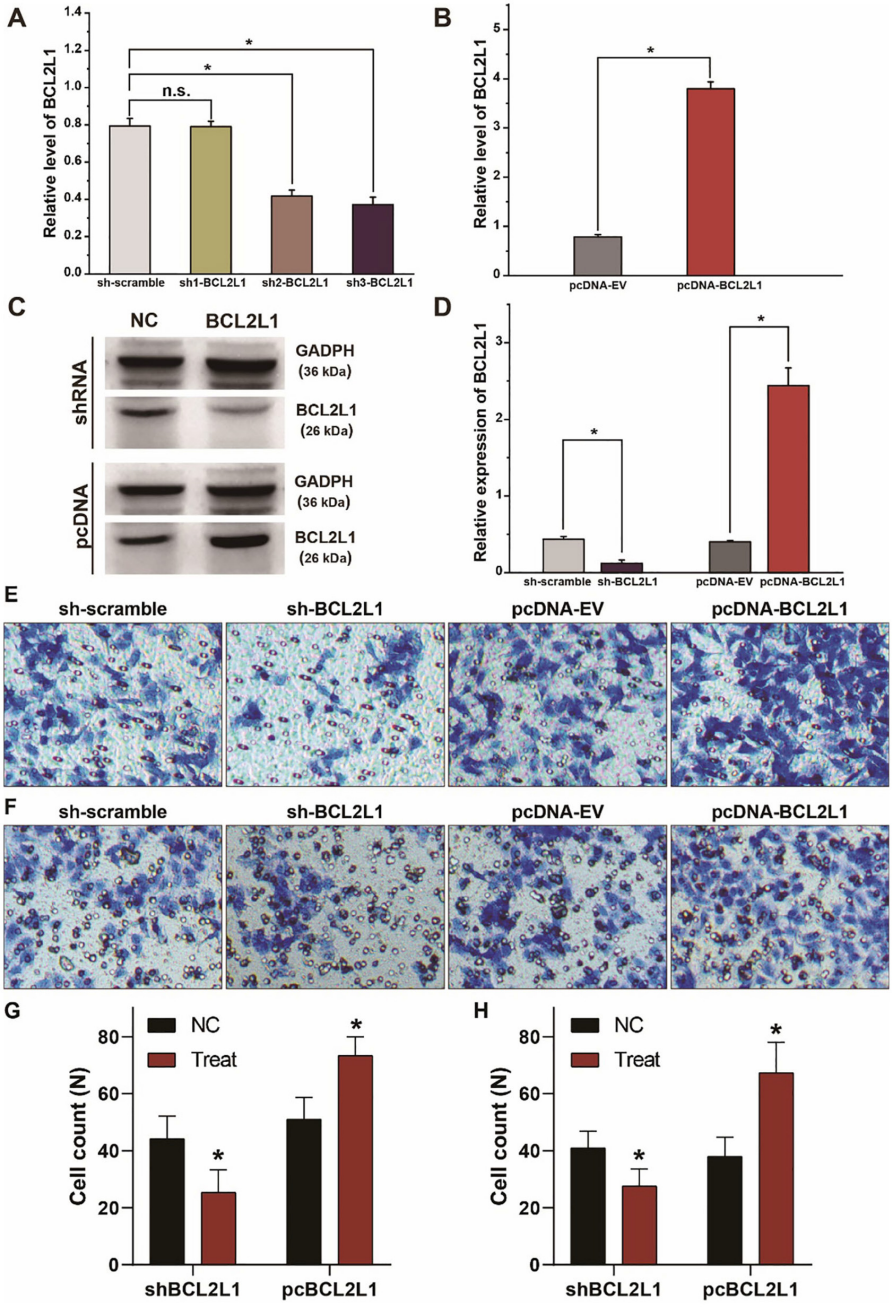
BCL2L1 modulates the migration and invasion of A549 lung cancer cells. **(A)** Examination of the knockdown efficiency of sh-BCL2L1 plasmid using RT-qPCR. **(B)** RT-qPCR assays of BCL2L1 in A459 lung cancer cells transfected with pc-BCL2L1 or negative control. **(C,D)** Western blot gels and protein expression analysis of BCL2L1 in A459 lung cancer cells transfected with shRNAs or pcDNAs. **(E,F)**, Transwell assays show the alterations of cell invasion **(E)** and migration **(F)** of A459 lung cancer cells. **(G,H)** Quantitative analysis of **(E,F)**. NC represents the negative control. ^*^P < 0.05 compared with the control group using the pooled variance *t*-test.

**Figure 5:**
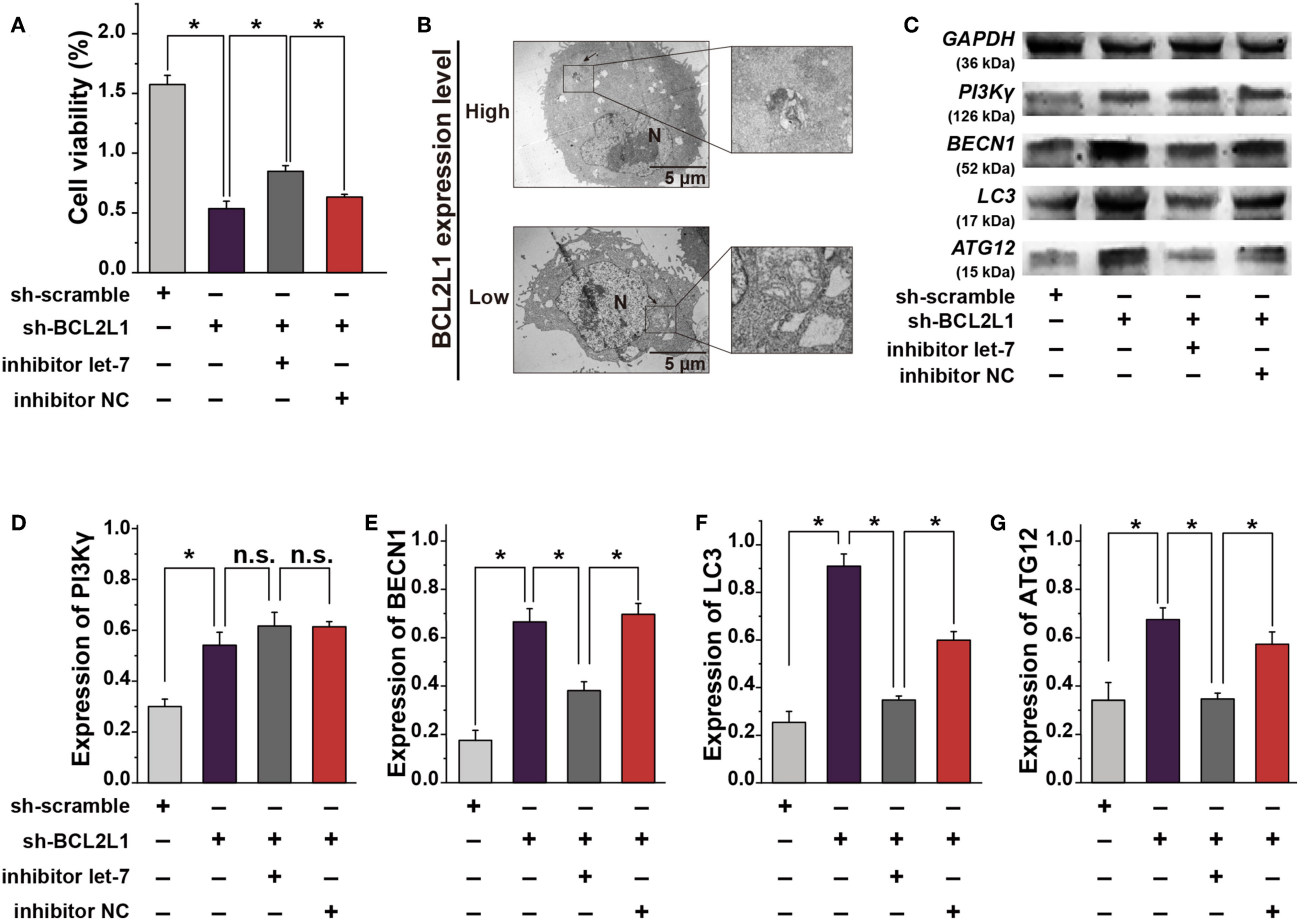
The crosstalk between let-7a-5p and BCL2L1 affects the autophagy and cellular viability of lung cancer cells. **(A)** CCK8 assays show the viability of A459 lung cancer cells. **(B)** Ultrastructural characteristics of A459 lung cancer cells captured by the transmission electron microscope. Arrows denote the autophagosomes. **(C)** Western blot gels of GAPDH, PI3Kγ, BECN1, LC3, ATG12. **(D–G)** Quatitative analysis of PI3Kγ, BECN1, LC3, ATG12. N, nucleus; Let-7, let-7a-5p; NC, negative control. ^*^P < 0.05 compared with the indicated group using the pooled variance *t*-test.

To explore the biological function of BCL2L1 in the proliferation, migration, and invasion of lung cancer cells, we designed and synthesized the plasmids of shRNAs and pcDNAs to silence or overexpress the expression of BCL2L1 in A549 lung cancer cells. As shown in RT-qPCR and western blot, the expression of BCL2L1 was downregulated after transfection of sh-BCL2L1, while it increased after transfection of pc-BCL2L1 (Figures 4A–D). We then performed transwell assays to evaluate the migration and invasion of A549 lung cancer cells. Silencing BCL2L1 attenuated the migration and invasion of A549 lung cancer cells, whereas overexpression of BCL2L1 elevated the migration and invasion of A549 lung cancer cells (Figures 4E–H). Meanwhile, we conducted rescue experiments by co-transfection of let-7a-5p inhibitors in sh-BCL2L1 transfected A549 lung cancer cells. Comparing with cells solely transfected with sh-BCL2L1, CCK8 assays showed significant recovery of cellular viability in A549 lung cancer cells that were co-transfected with let-7a-5p inhibitors (Figure 5A). Therefore, BCL2L1 is an import promoter in lung cancer cell proliferation, migration, and invasion.

### Inhibition of BCL2L1 Using Let-7a-5p Induces Autophagy in A549 Lung Cancer Cells

To investigate the mechanism that overexpression of let-7a-5p induces autophagy in A549 lung cancer cells (Figure 1B), we explored the ultrastructural characteristics of A549 lung cancer cells with different treatments. The transmission electron microscope showed that knockdown of BCL2L1 contributed to the formation of autophagosomes, and many cells were captured with damaged cellular membrane, blurred cytoplasm, or expanded dimensions, the undigested organelles in the autophagosomes could be clearly observed, the morphological integrity of these cells was destroyed. While overexpression of BCL2L1 inhibited the formation of autophagosomes, the cellular integrity was unaffected (Figure 5B). Additionally, we detected the expression of autophagy related biomarkers, including BECN1, PI3Kγ, ATG12, and LC3, which have been confirmed to be associated with the activation of autophagy in previous studies (13–16), and we found that knockdown of BCL2L1 promoted the expression of all these genes, while overexpression of BCL2L1 inhibited the expression of Beclin1, Atg12, and LC3 (Figures 5C–G). Besides, the cellular viability detected using CCK8 assays showed similar trend with the autophagy-related biomarkers (Figure 5A). Thus, these data suggest that BCL2L1 is a suppressor for initiating autophagy in A549 lung cancer cells.

### Effect of Let-7a-5p-BCL2L1 Crosstalk on the Apoptosis/Pyroptosis of A549 Lung Cancer Cells

**Figure 6:**
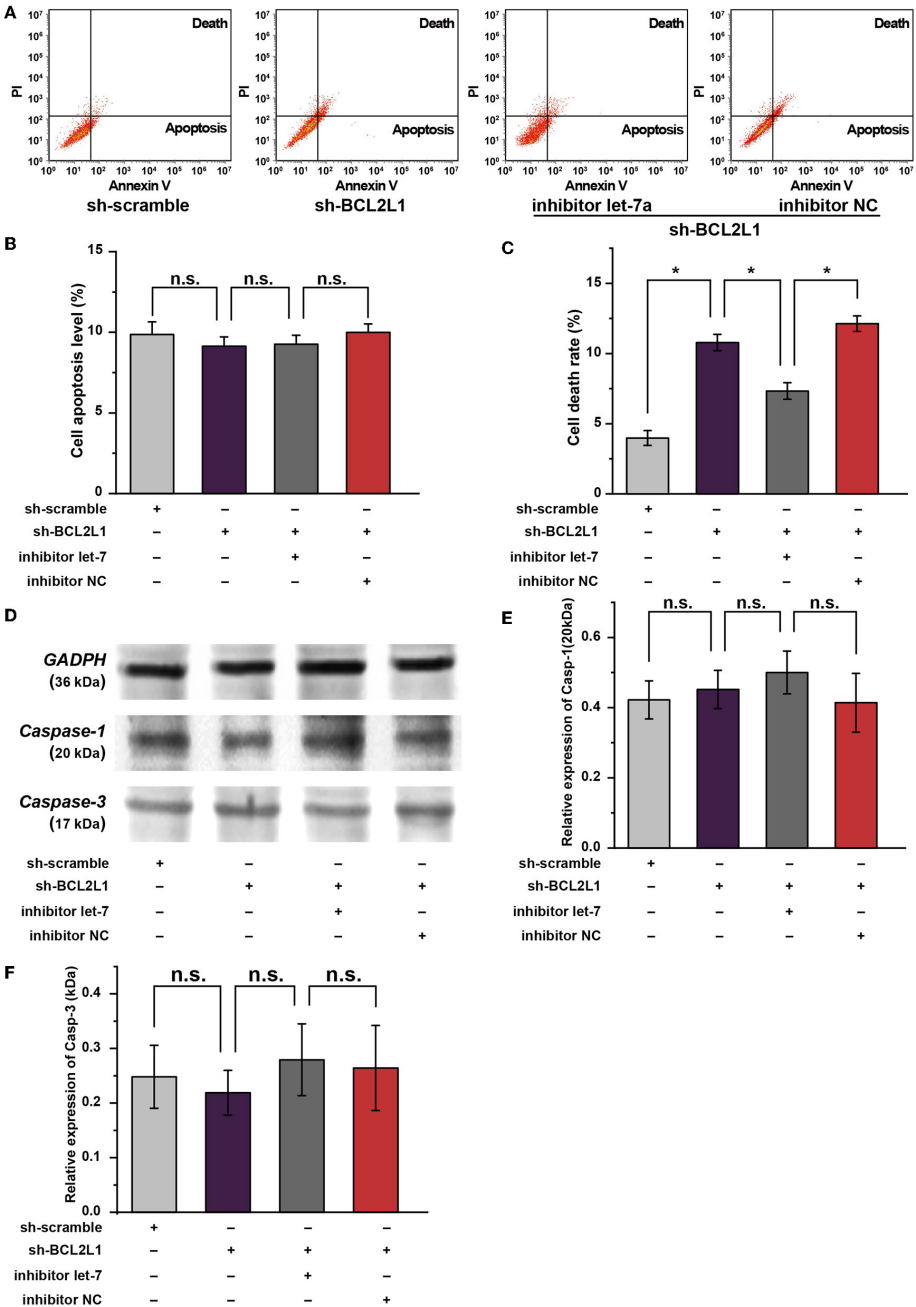
Role of let-7a-5p-BCL2L1 crosstalk in the apoptosis and pyroptosis of A549 lung cancer cells. **(A)** Results of flow cytometry showed the effects of let-7a-5p-BCL2L1 crosstalk on cell death and apoptosis of A459 lung cancer cells. **(B,C)** Quantitative analysis of cell death and apoptosis in A459 lung cancer cells. **(D–F)** Western blot membranes and protein expression analysis for biomarkers of pyroptosis and apoptosis. let-7, let-7a-5p; Casp-1, cleaved-Caspase-1; Casp-3, cleaved-Caspase-3; NC, negative control. ^*^P < 0.05 compared with the indicated group using the pooled variance *t*-test.

Apart from autophagy, available studies suggest that the apoptosis/pyroptosis alters the viability of A549 lung cancer cells (17, 18). Interestingly, BCL2 families are well-known anti-apoptosis factors or pyroptosis regulators (19, 20). To investigate the effect of BCL2L1 on the apoptosis/pyroptosis of A549 lung cancer cells, we conducted cellular flow cytometry to evaluate the cell death rate in different intervention groups of A549 lung cancer cells. As shown in Figure 6A, knockdown of BCL2L1 elevated the cell death rate, overexpression of BCL2L1 decreased the cell death rate, whereas the apoptosis rate was unaffected (Figures 6A–C). We then performed western blot to detect the protein biomarkers related to apoptosis and pyroptosis. Two biomarkers, cleaved-*Caspase-1* and cleaved-*Caspase-3*, were employed to indicate pyroptosis and apoptosis, respectively (21, 22). We found that these two protein biomarkers were unaffected by dysregulation of BCL2L1 (Figures 6D–F). These data demonstrate that aberrant expression of BCL2L1 alters the viability of lung cancer cells, while it does not change the apoptosis/pyroptosis of lung cancer cells.

### The Crosstalk of Let-7a-5p-BCL2L1 Alters the Migration of Lung Cancer Cells and the Expression of Lung Cancer Biomarkers *in vitro*

**Figure 7:**
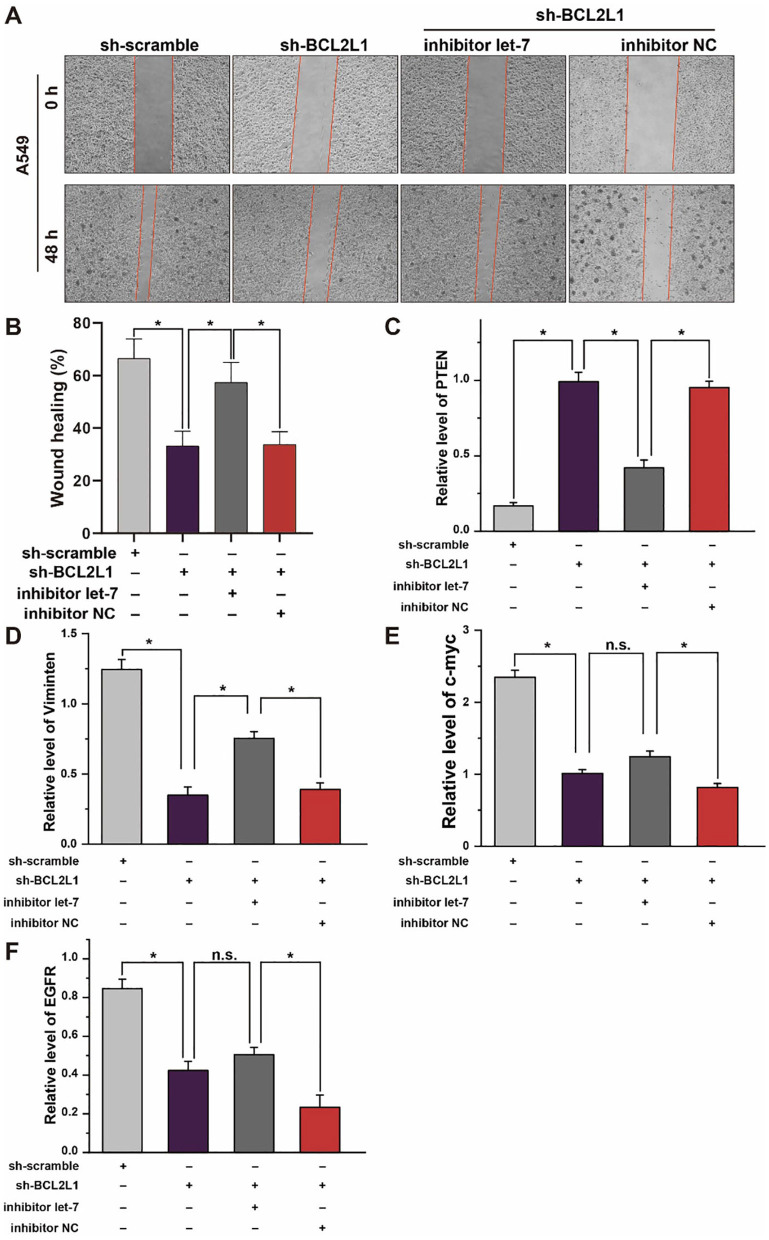
Effect of let-7a-5p-BCL2L1 crosstalk on lung cancer cells *in vitro*. **(A)** Wound healing assays exhibit the effect of let-7a-5p-BCL2L1 crosstalk on the migration of A459 lung cancer cells. **(B)** Quantitative analysis of **(A)**. **(C-F)** RT-qPCR analysis of lung cancer biomarkers. let-7, let-7a-5p; NC, negative control. ^*^P < 0.05 compared with the indicated group, using the pooled variance *t*-test.

To explore the effect of let-7a-5p-BCL2L1 crosstalk on the migration of A459 lung cancer cells, we conducted wound healing assays. As shown in Figures 7A,B, knockdown of BCL2L1 suppressed the wound healing capability of A549 lung cancer cells, while overexpression of BCL2L1 enhanced the cells' wound healing capability. In addition, the expression of PTEN, MYC, EGFR, and Vimentin, potential indicators associated with lung cancer patients survival period (23–26), was also detected in A549 lung cancer cells. Knockdown of BCL2L1 significantly attenuated the expression of MYC, EGFR, and Vimentin, while overexpression of BCL2L1 elevated their expressions (Figures 7D–F). In contrast, PTEN, the anti-tumor indicator, was upregulated after knockdown of BCL2L1 and downregulated in the recuse experiments (Figure 7C).

## Discussion

To investigate the role of let-7a-5p-BCL2L1 crosstalk in lung cancer, we performed functional annotation and signaling pathway enrichment for let-7a-5p. As shown in Figure 1 and Table S1, let-7a-5p and its 4 target genes were clustered into 2 groups of apoptosis and autophagy, among which BCL2L1, IGF1R, and MAPK8 were mainly involved in autophagy, while BCL2L1, FAS, and MAPK8 were predicted to be associated with apoptosis. Consistent with previous studies, BCL2L1, collaborating with VDAC2, could suppress autophagy and improve female fecundity (27). Autophagy mediated by MAPK8/9/10 could be repressed by Rabl3, leading to poor survival of non-small cell lung cancer (28). IGF1R has been proved to be regulated by let-7 families in T cell protection (29), and dysregulation of EGFR using chemically synthesized small interfering RNAs also lead to autophagy, in which the potential mechanisms are associated with the PI3Kγ/AKT/mTOR signaling pathway (30). On the other hand, BCL2L1, FAS, and MAPK8 are widely reported in cell apoptosis (31–33). Based on the epidemiology survey and Kaplan-Meier survival analysis among 2,437 lung cancer patients, the alteration of BCL2L1 affects the lung cancer patients' survival. Therefore, it is essential to deep investigate the role of BCL2L1 for understanding the carcinogenesis and development of lung cancer.

The dual-luciferase reporter assay was first adopted to examine the regulatory mechanism of let-7a-5p-BCL2L1 crosstalk. As shown in Figure 2, the luciferase activity was downregulated in cells that were treated with let-7a-5p mimics, while the luciferase activity was unaffected after inducing mutation in the seed region of BCL2L1, indicating that BCL2L1 could be inhibited by let-7a-5p. Indeed, the mediatory relationship between le-7 families and BCL2L1 has been demonstrated in previous studies. For example, overexpression of let-7c leads to BCL2L1 repression and induces apoptosis in liver cancer cells (34), and repression of BCL-XL by let-7c promotes ox-LDL induced endothelial apoptosis (35). However, to the best of our knowledge, the role of let-7a-5p-BCL2L1 crosstalk in lung cancer has not been reported.

In general, autophagy is beneficial for cell survival, and it is sometimes a must for cancer cells to adapt rapid growing conditions (36, 37). To reveal the role of let-7a-5p-BCL2L1 crosstalk on autophagy in lung cancer, we observed the ultrastructural changes of the A459 lung cancer cells and examined the cellular viability under different conditions. We found that overexpression of BCL2L1 led to autophagy suppression, but inadequate BCL2L1 promoted lung cancer cell migration and invasion. Considering the anti-apoptosis capability of BCL2 families, we assessed the apoptosis/pyroptosis of A549 lung cancer cells by detecting the expression of the cleaved *Caspase-3* and *Caspase-1*, respectively. The results of western blot showed that the expression of both protein biomarkers was unaffected by the aberrant expression of BCL2L1. All of which indicated that dysregulation of BCL2L1 could not initiate or block the apoptosis or pyroptosis biological process in A549 lung cancer cells, while autophagy induced by let-7a-5p-BCL2L1 crosstalk adversely affects lung cancer cell migration and invasion. Interestingly, several studies also found similar phenomenon with this study. For example, excessive autophagy induces neuron death in cerebral ischemia (38), and autophagy induced by oxidative stress in preeclampsia also led to the failure of trophoblast invasion and vasculature (39). Several more recent studies confirmed that autophagy is involved in X-linked myopathy (40, 41). However, several studies demonstrate that autophagy exhibits a tumor suppressing role in precancerous lesions, whereas it has a tumor-promoting role in full-blown cancer (42–44). Considering the dual role of autophagy, we detected the cell death using flow cytometry, in which the PI-positive cells were treated as dead cells. From the TEM images of BCL2L1 highly expressed A549 cells, we found the damaged cellular membrane and nuclear membrane, while the loss of cell membrane integrity is essential for PI entering nucleus and binding to DNA, so we conclude that autophagy induces the damage of cell membrane, which in turn lead to cell death. For the role of autophagy in lung cancer, it may be that excessive autophagy induces lung cancer cell death. In this study, we found that BCL2L1 is a suppressor of macroautophagy, so high expression of BCL2L1 reduces excessive autophagy in A549 cells, which is good for cell survival.

Collectively, our findings confirm that upregulation of let-7a-5p leads to lung cancer cell death, the underlying mechanism is associated with the autophagy burst induced by the inhibition of BCL2L1 through PI3Kγ signaling pathway, which provide emerging insight into the investigation of lung cancer carcinogenesis and development, and let-7a-5p, as an efficient BCL2L1 inhibitor, might be a useful target for lung cancer treatment.

## Methods

### Cell Culture and Intervention

The A549 lung cancer cells derived from human lung adenocarcinoma were obtained from ATCC Company (ATCC, Manassas, VA, USA) and maintained in high glucose complete Dulbecco's modified Eagle medium (DMEM) (Hyclone, Utah, USA) in a humidified atmosphere at 37°C and 5% CO_2_, the complete DMEM was composed of 10% fetal bovine serum (FBS), 100 μg/mL streptomycin, 100 U/mL penicillin and 2 mM glutamine. After being passaged for three generations, the A549 lung cancer cells obtained from the fourth generation were used for downstream experiments. When the A549 lung cancer cells growing up to 70% confluence of 60 mm dish, the chemically synthesized mimics or inhibitors of let-7a-5p were added to the serum-free medium. Keep incubating for 48 h, the A549 lung cancer cells were harvested for further use.

### Functional Annotation and Pathway Enrichment of Let-7a-5p

Let-7a-5p and its 4 target genes including BCL2L1, IGF1R, MAPK8, and FAS were employed to conduct functional annotation and pathway enrichment using R software version 3.6 for Windows based on databases of Gene Ontology (GO) and Kyoto Encyclopedia of Genes and Genomes (KEGG), in which 4 features of cellular component, molecular function, biological process, and signaling pathway were analyzed, respectively. Functional items that satisfied the conditions pre-set (P <0.05, false discovery rate (FDR) <0.01, fold change (FC) ≥ 2.0 or ≤ 0.5) were selected and clustered into different functional groups.

### Kaplan-Meier and Cox Hazards Regression Survival Analysis

Kaplan-Meier and Cox hazards regression survival analysis was performed for all target genes using the online database registered as KM plotter for lung cancer (available online at http://kmplot.com/analysis/). The database integrated 54,675 genes on survival using 10,461 cancer samples, including 2,437 lung cancer patients with a mean follow-up time of 49 months. The target genes related to patient survival in lung cancer were selected for downstream analysis.

### Plasmids Construction and Transfection

The mature sequences of let-7a-5p mimics, inhibitors, and their corresponding negative controls (listed in below) were designed and synthesized by Sangon Biotech (Shanghai, China). The inserted fragment was validated using DNA sequencing technology. Cell transfection and co-transfection were conducted using Lipo6000 (Beyotime, Shanghai, China). Firefly luciferase reporter plasmids containing 3′ untranslated region (UTR) of the BCL2L1 were purchased from AmyJet Scientific Inc. (Wuhan, China). The mutations were induced with the supposed target site of BCL2L1 3′-UTR using the QuickChange XL kit (Stratagene, La Jolla, CA, USA) according to the manufactures instructions. The primers were:

let-7a-5p mimic: 5′-UGAGGUAGUAGGUUGUAUAGUU-3′

Negative control: 5′-UUCUCCGAACGUGUCACGUTT-3′

let-7a-5p inhibitor: 5′-AACUAUACAACCUACUACCUCA-3′

Negative control : 5′-AGCUCCCAAGAGCCUAACCCGU-3′

For aberrant expression of BCL2L1, the A549 lung cancer cells were transfected with pcDNA3.1 plasmids and shRNAs, respectively. To overexpress BCL2L1, the full length of BCL2L1 cDNA was amplified and subcloned into pcDNA3.1; whereas 3 shRNAs were synthesized to knockdown the expression of BCL2L1. The empty pcDNA3.1 plasmid and a scrambled shRNA were used as negative controls, respectively. All plasmids were isolated and purified using the AxyPrep DNA Miniprep Kit (Axygen Scientific, CA, USA).

### Luciferase Reporter Plasmid Assay

The A549 lung cancer cells were plated in the 6-well plates with 80% confluence and transfected with 0.5 μg reporter plasmid along with 50 nmol let-7a-5p mimics, inhibitors or their corresponding controls, respectively. The pRL-CMV plasmid expressing Renilla luciferase was also transfected to each group as the internal control. After incubating for 24 h, the cells were harvested with the trypsin at the concentration of 0.25%, 37°C for 1 min. The fluorescence intensity of Firefly and Renilla was detected, and all values stained with Firefly were normalized with values of Renilla, each experiment was repeated with 5 samples in parallel.

### Migration and Invasion Assay

The protocols used to detect cell migration and invasion were previously reported (29). In brief, the motility of A549 lung cancer cells was measured using a Transwell apparatus. A549 lung cancer cells transfected with plasmids were seeded in the log phase of the Transwell apparatus, and the serum free medium was renewed 2 h later, the medium in the lower chamber was replaced with 10% FBS containing DMEM. For invasion assays, the filters of the Transwell chambers were coated with 30 μg Matrigel (BD Biosciences, San Jose, CA, USA). Keep incubating for 24 h in a standard cell incubator, the cells were harvested at the bottom of the Transwell apparatus, fix with 100% methanol for 2 min, stain in 0.5% crystal violet for 2 min, and rinse with PBS. Finally, the cells were subjected to an optical microscope (CKX41, Olympus, Tokyo, Japan). The cell migration and invasion were evaluated by randomly counting 5 fields of the stained cells.

### Protein Immunoblotting

Total protein of A549 lung cancer cells from different transfection groups was extracted and quantified. Then 40 μg protein was diluted in 20 μL volume of PBS and added to sodium dodecyl sulfate-polyacrylamide gel electrophoresis (SDS-PAGE) and transferred onto PVDF membranes (Millipore, Billerica, MA). After incubating in 5% non-fat milk at 4°C with rabbit anti-human primary antibody of BCL2L1, Caspase-1, Caspase-3, and GAPDH (Abcam, Cambridge, MA, USA) for 12 h, the protein coated membrane was washed with TBST for three times, 10 min per time. All membranes were then exposed to goat anti-rabbit secondary antibodies (Abcam, Cambridge, MA, USA). After washing for three times with TBST, 10 min per time, the membranes were subjected to Fluor Chem HD2Gel imaging system (Proteinsimple, San Jose, CA, USA) for signal measurement. All samples used in protein detection were repeated for five times in parallel.

### Real-Time Quantitative PCR (RT-qPCR)

The A549 lung cancer cells were harvested from different transfection groups, and the total mRNAs were then isolated. cDNAs were obtained using mRNA reverse kit (Sangon Biotech, Shanghai, China). The GAPDH was used as internal control. RT-qPCR was conducted using SYBR Green Method (Sangon Biotech, Shanghai, China) with StepOnePlus Real-Time PCR System (Applied Biosystems, Foster, CA, USA). The PCR primers were:

let-7a-5p: 5′- TGAGGTAGTAGGTTGTATAGTT-3′
BCL2L1 forward primer: 5′-GCATATCAGAGCTTTGAACAGG-3′
BCL2L1 reverse primer: 5′-GAAGGAGAAAAAGGCCACAATG-3′
BECN1 forward primer: 5′-ATCTAAGGAGCTGCCGTTATAC-3′
BECN1 reverse primer: 5′-CTCCTCAGAGTTAAACTGGGTT-3′
LC3 forward primer: 5′-CCTGGACAAGACCAAGTTTTTG-3′
LC3 reverse primer: 5′-GTAGACCATATAGAGGAAGCCG-3′
PTEN forward primer: 5′-GACCAGAGACAAAAAGGGAGTA-3′
PTEN reverse primer: 5′-ACAAACTGAGGATTGCAAGTTC-3′
EGFR forward primer: 5′-ACCCATATGTACCATCGATGTC-3′
EGFR reverse primer: 5′-GAATTCGATGATCAACTCACGG-3′
MYC forward primer: 5′-CGACGAGACCTTCATCAAAAAC-3′
MYC reverse primer: 5′-CTTCTCTGAGACGAGCTTGG-3′
PI3Kγ forward primer: 5′-CACACACTACATCAGTGGCTCAAAG-3′
PI3Kγ reverse primer: 5′-TCCAGCACATGAACGTGTAAACAG-3′
ATG12 forward primer: 5′-AACAAAGAAATGGGCTGTGG-3′
ATG12 reverse primer: 5′-TTGCAGTAATGCAGGACCAG-3′
Vimentin forward primer: 5′-GAGAACTTTGCCGTTGAAGC-3′
Vimentin reverse primer: 5′-GCTTCCTGTAGGTGGCAATC-3′
GAPDH forward primer: 5′-ACCCAGAAGACTGTGGATGG-3′
GAPDH reverse primer: 5′-TCTAGACGGCAGGTCAGGTC-3′.

### Wound Healing Assays

An equal number of A549 lung cancer cells from different transfection groups were seeded onto 6-well plates, and then they were incubated at a standard atmosphere for 24 h. A wound was scraped into the middle region of the cell growing area with a plastic 200 μL tip and washed with PBS for three times. At the time point 0 and 48 h, the wounds were captured using an optical microscope (CKX41, Olympus, Tokyo, Japan). The distance of the scratches was measured using Photoshop CS5.0 for Windows (Adobe Systems, San Jose, CA, USA) and the healing ability of A549 lung cancer cells was assessed.

### Transmission Electron Microscope

The A549 lung cancer cells harvested from each transfection group were fixed in 2.5% glutaraldehyde and 0.1 M cacodylate buffer (pH 7.4) for 2 h. After washing with PBS, the cells were post-fixed in 2% osmium tetroxide. Then, the cell pellets were embedded in Epon resin. The ultra-structures of A549 lung cancer cells were observed under the transmission electron microscope (Hitachi, Tokyo, Japan) at an acceleration voltage of 80 kV.

### Cell Flow Cytometry

The A549 lung cancer cells with different transfections were seeded onto 6-well plates, keep growing up to 80% confluence, the cells were harvested by trypsin enzyme digesting. Then the cells were stained with PI/Annexin V using Annexin V-FITC Apoptosis Detection Kit (Beyotime Biotechnology, Shanghai, China) and subjected to BD Accuri C6 flow cytometry (BD Medical Technology, Franklin Lakes, NY, USA), and the data were analyzed using FCS Express 6.0 (De Novo Software, Glendale, CA, USA).

### Statistical Analysis

All data were analyzed using the SAS software package version 9.2 for Windows (SAS Institute Inc., Cary, NC, USA). The continuous variables of normal distribution were described as mean ± standard deviation. The differences between any two groups were compared using the pooled variance *t*-test. A *P* < 0.05 was considered statistically significant unless otherwise indicated.

## Data Availability

All datasets generated for this study are included in the manuscript and/or the Supplementary Files.

## Author Contributions

LZ and SYa designed the study and wrote the manuscript. SD, SYa, SYu, TY, and LZ performed the work. SD analyzed the data and interpreted the results. All authors have read and approved the final version of the manuscript.

### Conflict of Interest Statement

The authors declare that the research was conducted in the absence of any commercial or financial relationships that could be construed as a potential conflict of interest.

## Abbreviations

AKT: Protein kinase B
BCL2L1: Bcl-2-like protein 1
CI: Confidence intervals
DMEM: Dulbecco's modified Eagle medium
FBS: Fetal bovine serum
FC: Fold change
FDR: False discovery rate
GO: Gene Ontology
HR: Hazard ratio
KEGG: Kyoto Encyclopedia of Genes and Genomes
KM: Kaplan-Meier
MAPK8: Mitogen-activated protein kinase 8
PI3K_γ_: Phosphatidylinositol-4,5-bisphosphate 3-kinase gamma
PTEN: Phosphatase and tensin homolog
RT-qPCR: Real-time quantitative PCR
SDS-PAGE: Sodium dodecyl sulfate-polyacrylamide gel electrophoresis
UTR: Untranslated region.
