# Corrigendum: Exogenous let-7a-5p induces A549 lung cancer cell death through BCL2L1-mediated PI3Kγ signaling pathway

**DOI:** 10.3389/fonc.2024.1513956

**Published:** 2024-12-02

**Authors:** Shuyin Duan, Songcheng Yu, Teng Yuan, Sanqiao Yao, Lin Zhang

**Affiliations:** ^1^ Key Laboratory of Birth Regulation and Control Technology of National Health Commission of China, Shandong Maternal and Child Health Care Hospital, Jinan, China; ^2^ School of Public Health, Zhengzhou University, Zhengzhou, China; ^3^ College of Jitang, North China University of Science and Technology, Tangshan, China; ^4^ School of Public Health, Xinxiang Medical University, Xinxiang, China; ^5^ School of Public Health and Management, Weifang Medical University, Weifang, China

**Keywords:** lung cancer, macrophage, exosome, BCL2L1, let-7a-5p, autophagy

In the published article, errors appeared in **Figures 3B**, **C**, **4E**, **F**, and **7A**, **B**. During the transwell assay and scratch test procedures, we used the equipment’s default image naming system for batch exports, which led to difficulties in distinguishing between intervention groups during image selection and resulted in incorrect image placement. Given that a significant amount of time has elapsed since publication, the original data associated with these results are no longer available. We therefore carried out independent repeat experiments and achieved outcomes consistent with the initial findings. As a result, the relevant images and their quantitative data in **Figures 3B–E**, **4E–H**, and **7A**, **B** have been updated.

The corrected **Figure 3** and its caption are provided below.

**Figure 3:**
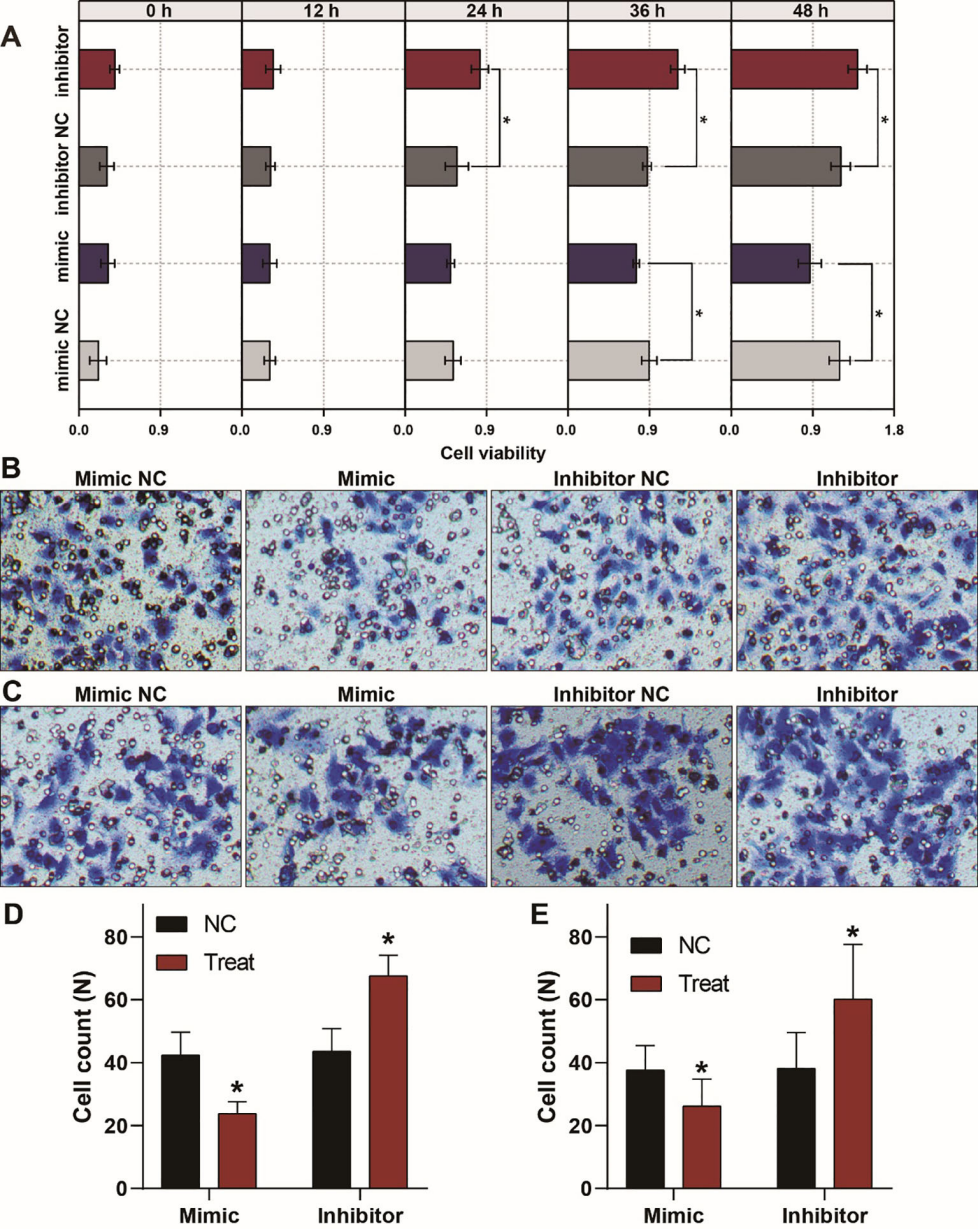
Aberrant expression of let-7a-5p alters the growth, migration, and invasion of A459 lung cancer cells. **(A)** CCK8 assays show that let-7a-5p suppresses the growth of A549 lung cancer cells. **(B, C)** Transwell assays demonstrate that let-7a-5p inhibits the invasion **(B)** and migration **(C)** of A549 lung cancer cells. **(D, E)** Quantitative analysis of **(B, C)**. NC represents the negative control. ^*^ *p* < 0.05 compared with the control group, using the pooled variance *t*-test.

The corrected **Figure 4** and its caption are provided below.

**Figure 4:**
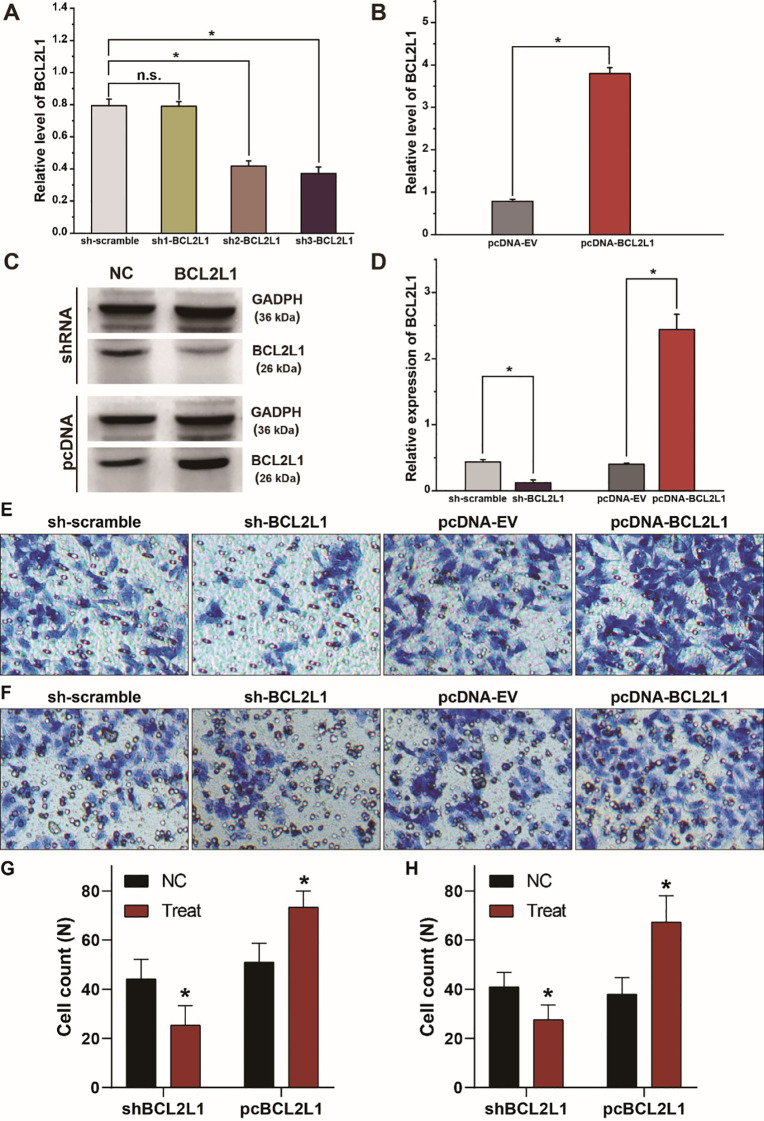
BCL2L1 modulates the migration and invasion of A549 lung cancer cells. **(A)** Examination of the knockdown efficiency of the sh-BCL2L1 plasmid using RT-qPCR. **(B)** RT-qPCR assays of BCL2L1 in A459 lung cancer cells transfected with pc-BCL2L1 or negative control. **(C, D)** Western blot gels and protein expression analysis of BCL2L1 in A459 lung cancer cells transfected with shRNAs or pcDNAs. **(E, F)**, Transwell assays show the alterations of cell invasion **(E)** and migration **(F)** of A459 lung cancer cells. **(G, H)** Quantitative analysis of **(E, F)**. NC, negative control. ^*^ *p* < 0.05 compared with the control group using the pooled variance *t* test.

The corrected **Figure 7** and its caption are provided below.

**Figure 7:**
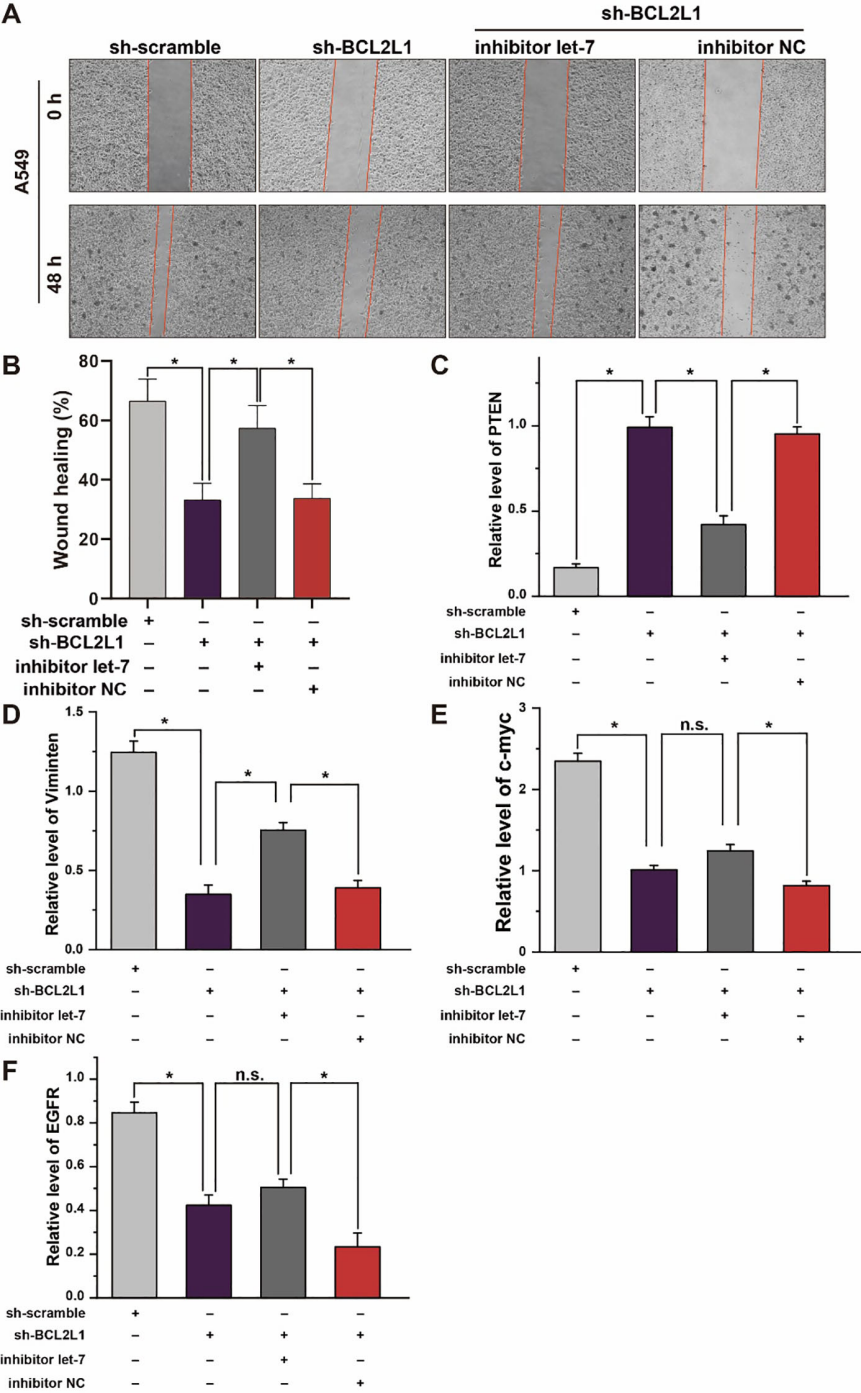
Effect of let-7a-5p-BCL2L1 crosstalk on lung cancer cells *in vitro*. **(A)** Wound healing assays exhibit the effect of let-7a-5p-BCL2L1 crosstalk on the migration of A459 lung cancer cells. **(B)** Quantitative analysis of **(A)**. **(C–F)** RT-qPCR analysis of lung cancer biomarkers. let-7, let-7a-5p; NC, negative control. ^*^ *p* < 0.05 compared with the indicated group, using the pooled variance *t*-test.

The authors apologize for these errors and confirm that this does not change the scientific conclusions of the. The original article has been updated.

