# Supplementary material for: Exogenous Let-7a-5p Induces A549 Lung Cancer Cell Death Through BCL2L1-Mediated PI3Kγ Signaling Pathway
Source: Front Oncol. 2019 Aug 23;9:808. doi: 10.3389/fonc.2019.00808 (PMC6716507; doi:10.3389/fonc.2019.00808)
Supplement: Supplementary file 1 [file Table_1.DOCX]

Supplemental information for

Macrophage-derived let-7a-5p triggers lung cancer cell death through

targeting BCL2L1-mediated PI3K signaling

Shuyin Duan, Songcheng Yu, Teng Yuan, Sanqiao Yao, Lin Zhang

This PDF includes:

Supplemental Table S1

**Supplemental Tables**

**Table S1.** Functional annotation of let-7a-5p in lung cancer.

| GOID | GOTerm | P Value | Genes |
| --- | --- | --- | --- |
| Autophagy |  |  |  |
| GO:0004140 | Autophagy | 1.10E-06 | BCL2L1, IGF1R, MAPK8 |
| GO:0004210 | Apoptosis | 1.35E-06 | BCL2L1, FAS, MAPK8 |
| GO:0004215 | Apoptosis | 1.64E-05 | BCL2L1, MAPK8 |
| GO:0004914 | Progesterone-mediated oocyte maturation | 1.41E-04 | IGF1R, MAPK8 |
| GO:0005212 | Pancreatic cancer | 6.25E-05 | BCL2L1, MAPK8 |
| GO:0090559 | regulation of membrane permeability | 1.18E-04 | BCL2L1, MAPK8 |
| GO:0046902 | regulation of mitochondrial membrane permeability | 1.05E-04 | BCL2L1, MAPK8 |
| Apoptosis |  |  |  |
| GO:0004210 | Apoptosis | 1.35E-06 | BCL2L1, FAS, MAPK8 |
| GO:0004215 | Apoptosis | 1.64E-05 | BCL2L1, MAPK8 |
| GO:0005212 | Pancreatic cancer | 6.25E-05 | BCL2L1, MAPK8 |
| GO:0090559 | regulation of membrane permeability | 1.18E-04 | BCL2L1, MAPK8 |
| GO:0038034 | signal transduction in absence of ligand | 8.14E-05 | BCL2L1, FAS |
| GO:0071260 | cellular response to mechanical stimulus | 1.00E-04 | FAS, MAPK8 |
| GO:0046902 | regulation of mitochondrial membrane permeability | 1.05E-04 | BCL2L1, MAPK8 |
| GO:0034198 | cellular response to amino acid starvation | 2.42E-05 | FAS, MAPK8 |
| GO:0097192 | extrinsic apoptotic signaling pathway in absence of ligand | 8.14E-05 | BCL2L1, FAS |
| GO:1904019 | epithelial cell apoptotic process | 1.47E-04 | BCL2L1, FAS |
| GO:0008625 | extrinsic apoptotic signaling pathway via death domain receptors | 1.32E-04 | BCL2L1, FAS |
| GO:0097284 | hepatocyte apoptotic process | 3.73E-06 | BCL2L1, FAS |
| GO:2001239 | regulation of extrinsic apoptotic signaling pathway in absence of ligand | 3.95E-05 | BCL2L1, FAS |
| GO:1902041 | regulation of extrinsic apoptotic signaling pathway via death domain receptors | 7.27E-05 | BCL2L1, FAS |
| GO:1902042 | negative regulation of extrinsic apoptotic signaling pathway via death domain receptors | 2.67E-05 | BCL2L1, FAS |
